# Facile fabrication of next-generation sustainable brick and mortar through geopolymerization of construction debris

**DOI:** 10.1038/s41598-024-61688-x

**Published:** 2024-05-13

**Authors:** Hamed Rahimpour, Alireza Babaeian Amini, Fatemeh Sharifi, Ahmad Fahmi, Sahar Zinatloo-Ajabshir

**Affiliations:** 1 Department of Civil Engineering, University of Tabriz, Tabriz, East Azerbaijan Iran; 2 Department of Civil Engineering, University of Bonab, Bonab, Iran; 3 Department of Chemical Engineering, University of Bonab, P.O. Box. 5551395133, Bonab, Iran

**Keywords:** Geopolymer, Alkaline solution, Sodium hydroxide, Water glass, Construction debris, Environmental sciences, Engineering, Materials science

## Abstract

Waste from construction and demolition (also known as CDW) is one of the most harmful environmental issues. This study's primary goal is to produce new mortar and brick materials from recycled concrete powder (RCP) and recycled brick powder (RBP), two of the most popular CDW. Geopolymeric mortar and brick samples were produced by passing RCP and RBP through sieve No. 50 (with sand filler if necessary) and combining them with an alkaline solution made of water glass (WG) and NaOH. In this study, the mixture was then cured for three days at 80 °C in an oven. The effects of filler, RBP amount, WG amount, and the concentration of NaOH alkaline solution on the samples’ strength were examined. Additionally, XRF and SEM/XRD tests were performed to verify the materials' composition and microstructure. The mechanical strength of the samples showed an increase with the increase of RCP values, so the brick sample with filler showed the highest compressive strength, measuring 59.53 MPa. The study's samples exhibited strong mechanical properties. Additionally, all of the bricks' water absorption fell within the standard range. In summary, according to different standards, both waste concrete and waste brick can be used to produce geopolymer materials especially bricks for construction and paving purposes.

## Introduction

One of the most popular and widely utilized building materials worldwide is concrete. Its strength, durability in a variety of environments, beauty, and affordability have made it a desirable material in the construction industry. The raw materials for concrete are easily accessible; Therefore, its production is simple and economical. However, the cement industry emits 7–10% of the world's CO_2_ each year^1–6^, and the production of one ton of cement requires 4 GJ of energy^7^. The annual production of ordinary Portland cement (OPC) concrete around the world exceeds 14 billion cubic meters^8^, and a major concern about this large-scale use of OPC is the significant CO_2_ emissions associated with cement production. In other words, to produce one ton of OPC, about one ton of CO_2_ is released into the atmosphere, and 110 kWh of energy and 60–130 kg of liquid fuel or a similar substance must be used to raise the temperature above 1000 °C, which is required for calcination and clinker production in cement plants^9^. After cement, brick is the most commonly utilized construction material. The conventional method, which produces the majority of bricks, requires the kiln temperature to rise to around 1000 °C in order to produce bricks with the appropriate strength. This results in increased fuel consumption, greenhouse gas emissions, the depletion of energy supplies, and harm to the environment.

The main cause of the rise of waste clay brick, like with waste concrete, is the expansion in construction and destruction resulting from urban redevelopment. The production of CDW has therefore increased environmental concerns in this industry. Consequently, using new materials and not recycling CDW leads to pollution and increased costs for both manufacture and disposal. In order to prevent future issues, in-depth research on CDW use is necessary as industry grows. CWDs contribute to around 30% of the waste produced worldwide^10^, which is a significant amount. Scientists estimate that recycling CDW significantly reduces energy consumption and carbon emissions^11–13^. Waste concrete makes up 67.5% of the 600 million tons of CDW produced in the United States each year; waste asphalt makes up 17.5%, wood waste makes up 6.8%, and waste brick makes up 2%^14^. Furthermore, CDW makes for 30–40% of all municipal waste produced in China^15^ and by 2026, 4 billion tons of CDW are expected to be produced there^16^. One-third of the waste produced in Europe is classified as CDW and Britain accounts for 100 million tons^17^. India generates 150 million tons of CDW yearly, with Southeast Asian countries producing over 472 million tons^18^. In terms of waste brick production, China generates 0.4 billion tons of waste bricks yearly^19^, whereas India produces 32% of its entire CDW in form of waste bricks^20,21^. Furthermore, between 2014 and 2012, the United States produced almost 44 million tons of waste bricks^22^. As a result, disposing of waste concrete and waste bricks requires a large landfill, which has negative effects on the environment and the economy. Therefore, recycling and reprocessing of these wastes can be a suitable solution.

In this regard, attempts have been made to utilize CDW as RCP and RBP in the production of concrete to reduce the negative environmental impacts of CDW^23–25^. Several studies have shown that substituting part of the OPC in concrete with RCP and RBP reduces the compressive strength of the concrete^26–28^. Consequently, it is reasonable to conclude that recycling RBP and RCP as OPC alternative or substitution has minimal effect on reducing CO_2_ emissions in the cement industry; as a result, the environmental implications of CDW cannot be successfully controlled in this away.

Based on the aforementioned circumstances, sustainable development is defined as the type of development that satisfies current needs without jeopardizing those of future generations^29^. In this way, scientists are trying to substitute eco-friendly materials with OPC in an effort to lessen the environmental risks associated with its manufacture. Thus, geopolymer and other next-generation green construction materials can serve as suitable substitutes for OPC. Numerous studies in this field showed that geopolymers are environmentally friendly and sustainable since they can be manufactured from a variety of waste materials^30–34^. Geopolymer can be produced from any siliceous and aluminous substance that reacts in an alkaline environment to produce polymer chains and linked networks^35–37^. As a result, pozzolan—active aluminosilicate sources—and alkaline compounds undergo hydrothermal treatment to create alkali-activated products^38^.

Given that OPC requires a lot of energy^20,39^ and produces a great deal of CO_2_ during production process, its use is not environmentally justified^40^. Thus, geopolymerization of aluminosilicate materials is a sustainable process for producing environmentally friendly bricks, mortar, and concrete^41^. However, researchers have used fly ash^42^, bagasse ash^43^, bottom ash^44^, rice husk ash^45^, copper mine tailings^46^, GGBFS^47^, and kaolin^48^ to make geopolymer bricks. Researchers were also interested in geopolymers made with RBP and RCP^49,50^. Their study included investigations into self-healing effect^51,52^, thermal insulation^53^, 3D print^54,55^ and resistance to temperature fluctuations^56,57^. Furthermore, more favorable results were obtained when CDW was recycled through geopolymerization than when used as a partial or full OPC replacement.

Over the next decades, a large number of concrete structures will reach the end of their service lives and will be demolished to make way for new construction. Additionally, this issue has been compounded by recent earthquakes that have occurred in various parts of the world. The large-scale demolition of concrete structures ultimately creates large amounts of CDW. These CDWs are expensive to manage and dispose of, and they also cause a secondary crisis in which large amounts of waste end up in the environment^58,59^. On the other hand, these CDWs can serve as raw materials for the production of recycled building materials. As a result, by recycling CDWs to produce new materials, the problems of waste accumulation in the environment and supplying raw materials for the construction are resolved. According to the above contents, a significant part of the CDW consists of concrete and bricks, which various researchers have previously studied separately for the recycling of clay bricks and concrete. However, there are substantial difficulties and costs associated with the recycling process when it comes to sorting construction debris and separating concrete, bricks or blocks from CDW.

The primary objective of this study is to introduce a novel approach for the recycling of waste concrete and waste brick, which comprise the majority of CDW. The foundation of this process is the use of these materials as an input to make geopolymer materials. Furthermore, this study investigates, for the first time, the feasibility of recycling waste concrete and waste brick without the requirement for a costly separation procedure. The expansion of the application of this method in the construction industry can have a significant effect on the economic efficiency of recycling CDW. This study has also looked at the repurposing of brick and mortar from waste concrete and waste brick.

## Materials and methods

### Materials

In this study, waste bricks and concrete were crushed with a jaw crusher and were then passed through sieve No. 50 (300 µm) to get RBP and RCP, which were then utilized as binders to make brick samples and geopolymer mortar. Moreover, an activating alkaline solution comprising an industrial sodium hydroxide solution (NaOH with 98% purity) and sodium silicate solution (water glass with SiO_2_/Na_2_O:3) was utilized. Additionally, sand that passed through sieve No. 4 (4.75 µm) and remained on sieve No. 8 (2.36 µm) was employed in the samples that included filler. RBP was also used in previous studies to create geopolymer samples since it contains pozzolanic aluminosilicate compounds^49,60^. Furthermore, RCP can be utilized to alter the properties of geopolymer samples—which will be covered in depth in this study—because of its high calcium concentration.

### Preparation of geopolymer mortar and brick

**Figure 1:**
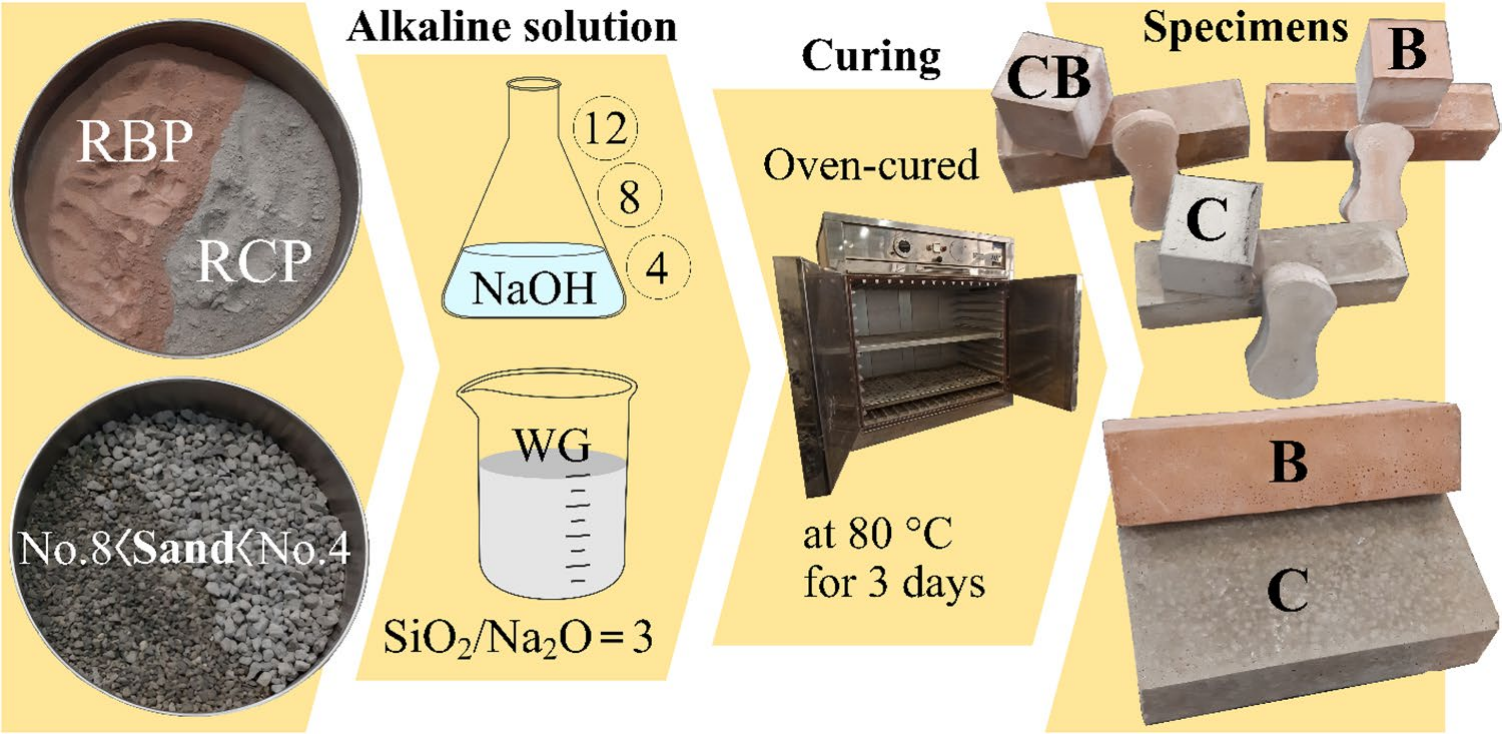
An overview of the raw ingredients, curing, color, and overall manufacturing process of the resulting geopolymer samples.

NaOH solution, water glass (WG), RBP derived from waste brick (grade SW clay brick), and RCP obtained from waste concrete (structural concrete with a minimum compressive strength of 20 MPa, grade M20) were utilized to produce geopolymer samples. In order to prepare NaOH solutions, varying amounts of sodium hydroxide pellets (AR grade) were dissolved in water to provide solutions with concentrations of 4, 8, and 12. The resulting NaOH solutions were then combined with WG at mass ratios of WG/NaOH: 1 and 2 (R1 and R2) then kept at room temperature for a full day. Next, in accordance with Table 2, the electric mortar mixer was filled with 100 and 50 wt.% of recycled powders, and the dry ingredients were blended to create a homogenous mixture (filler was added at this step if necessary). After that, the alkaline solution was gradually added and mixed for 5 min. The resulting paste was then poured into molds measuring 5 × 5 × 5 cm, 4 × 4 × 16 cm, and briquettes and were placed vibrating table for 30 s to remove air bubbles contained therein. It should be noted that the mass ratio of filler/pozzolan in the samples that contained filler was equal to 1. Finally, the samples were placed in the oven at a temperature of 80 °C and after 2 h, the samples were removed from the molds and kept in the oven for 3 days and then removed from the oven and cooled at room temperature until the relevant tests were done. It should be noted that the mix design of the brick samples was similar to that of the cubic mortar, except that the size of the brick molds was 5 × 10 × 20 cm. Figure 1 depicts the overall process of producing the geopolymer samples that were discussed above. The mix design for each kind of brick and mortar is given as well in Table 2. For every kind of specimen (compressive, flexural, and tensile), three samples were prepared. This mix design is consistent with the experiences obtained through earlier research^60–63^.

When closely examining Fig. 1 and contrasting it with Table 2, it is evident that the color tends to be orange for samples with a greater amount of RBP (B samples) and gray for samples with a higher amount of RCP (C samples).

### Characterization of geopolymer specimens

The compressive strength of the geopolymer samples with and without filler at the ages of 7- and 28-day were evaluated according to the ASTM C39^64^. In addition, flexural strength and tensile strength were evaluated only for filler-free mortars, which were according to ASTM C348-21^65^ and ASTM C307-23^66^, respectively.

To evaluate the water absorption of the geopolymer mortar samples, the ASTM C1585^67^ standard was followed. This involved weighing the samples dry and then immersing them in water for 30 min to allow water to run through all surfaces. They were subsequently pulled out of the water, rubbed dry with a towel, and their mass was once more measured. Additionally, brick samples were also subjected to water absorption in accordance with ASTM C642^68^, which involved weighing the bricks in a dry state before they were boiled for five hours in a water container and left there for fourteen hours to drop the sample's temperature down to 20–25 °C. Then the bricks were weighed to calculate their water absorption.

**Figure 2:**
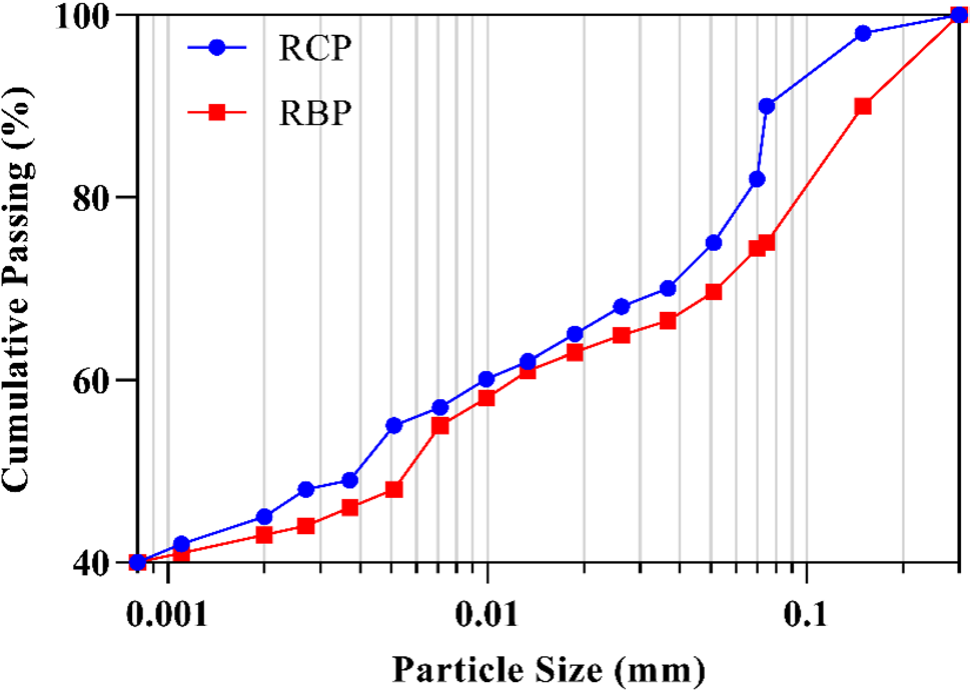
Particle size distributions of RCP and RBP.

An XRF test was performed on RBP and RCP to determine their chemical composition. The results are shown in Table 1 and are mostly composed of aluminosilicate components. Needless, both RBP and RCP have high calcium contents. Additionally, Fig. 2 illustrates the particle size distribution, with RCP and RBP having median particle sizes (d50) of 0.0037 and 0.0051 mm, respectively.

**Table 1:** Chemical composition of RBP and RCP (wt%). *LOI: Loss on ignition.

Sample type	SiO_2_	Al_2_O_3_	Fe_2_O_3_	CaO	Na_2_O	K_2_O	MgO	MnO	TiO_2_	P_2_O_5_	LOI*
RBP	42.40	10.68	5.50	14.45	2.42	1.75	4.83	0.11	0.57	0.26	1.72
RCP	41.37	8.59	4.53	24.01	3.46	1.32	3.00	0.07	0.38	0.30	11.94

SEM/EDX analysis was used to identify the microstructure of the crushed X-12-1 samples from the compressive strength test, as Table 2 illustrates. For this purpose, the MIRA3 FEG-SEM device made by Tescan company, Czech Republic was used, which has Field Emission and is appropriate for non-conductive substances such as mortar and concrete. It can also analyze samples qualitatively (types of components and phases that make up the substance) and quantitatively (amount and quantity of elements). As a result, crushed samples were utilized for SEM analysis to show the materials' non-polished and original structure. XRD analysis was carried out on RCP, RBP, and X-12-1 samples with a Tongda TD-3700 device, in China, utilizing a copper X-ray lamp anode and Ka_1_ copper radiation with a frequency of 1.5406 Angstroms as an X-ray source with a 2θ angle varying from 10 to 80°. For specimen preparation, the back loading technique was used^69^. The analysis accuracy of this device is 0.02 degrees per 0.5 s, and the voltage and current used are 30 kV and 20 mA, respectively. Furthermore, a flow table was used to conduct the flow test in accordance with the ASTM C1437^70^.

**Table 2:** Mix design and Chemical composition of geopolymer specimens.

Specimen	RCP (wt%)	RBP (wt%)	NaOH (M)	R^a^	l/b^b^	with filler			
						Si/Al	Na/Si	Na/Al	Ca/Si
RCP	100	0	0	0	0	4.3	NA	NA	4.16
RBP	0	100	0	0	0	2.44	0.7	0.18	0.75
C-12-1^c^	100	0	12	1	0.4	3.06	0.37	1.12	0.41
CB-12-1^d^	50	50	12	1	0.45	2.18	0.53	1.15	0.63
B-12-1^e^	0	100	12	1	0.5	3.93	0.9	3.57	0.27
C-8-1	100	0	8	1	0.4	–	–	–	–
CB-8-1	50	50	8	1	0.45	–	–	–	–
B-8-1	0	100	8	1	0.5	–	–	–	–
C-4-1	100	0	4	1	0.4	–	–	–	–
CB-4-1	50	50	4	1	0.45	–	–	–	–
B-4-1	0	100	4	1	0.5	–	–	–	–
C-12-2	100	0	12	2	0.45	–	–	–	–
CB-12-2	50	50	12	2	0.5	–	–	–	–
B-12-2	0	100	12	2	0.55	–	–	–	–
C-8-2	100	0	8	2	0.45	–	–	–	–
CB-8-2	50	50	8	2	0.5	–	–	–	–
B-8-2	0	100	8	2	0.55	–	–	–	–
C-4-2	100	0	4	2	0.45	–	–	–	–
CB-4-2	50	50	4	2	0.5	–	–	–	–
B-4-2	0	100	4	2	0.55	–	–	–	–

a: Water glass/NaOH b: l/b: Liquid to Binder ratio (ratio of alkaline solution to recycled powders) c: C-12-1 Represents a specimen made of RCP at 12M NaOH concentration and R1. d: CB-12-1 Represents a specimen made of a blend mix of RCP and RBP at 12M NaOH concentration and R1. e: B-12-1 Represents a specimen made of RBP at 12M NaOH concentration and R1.

## Results and discussion

### Mechanical strength

**Figure 3:**
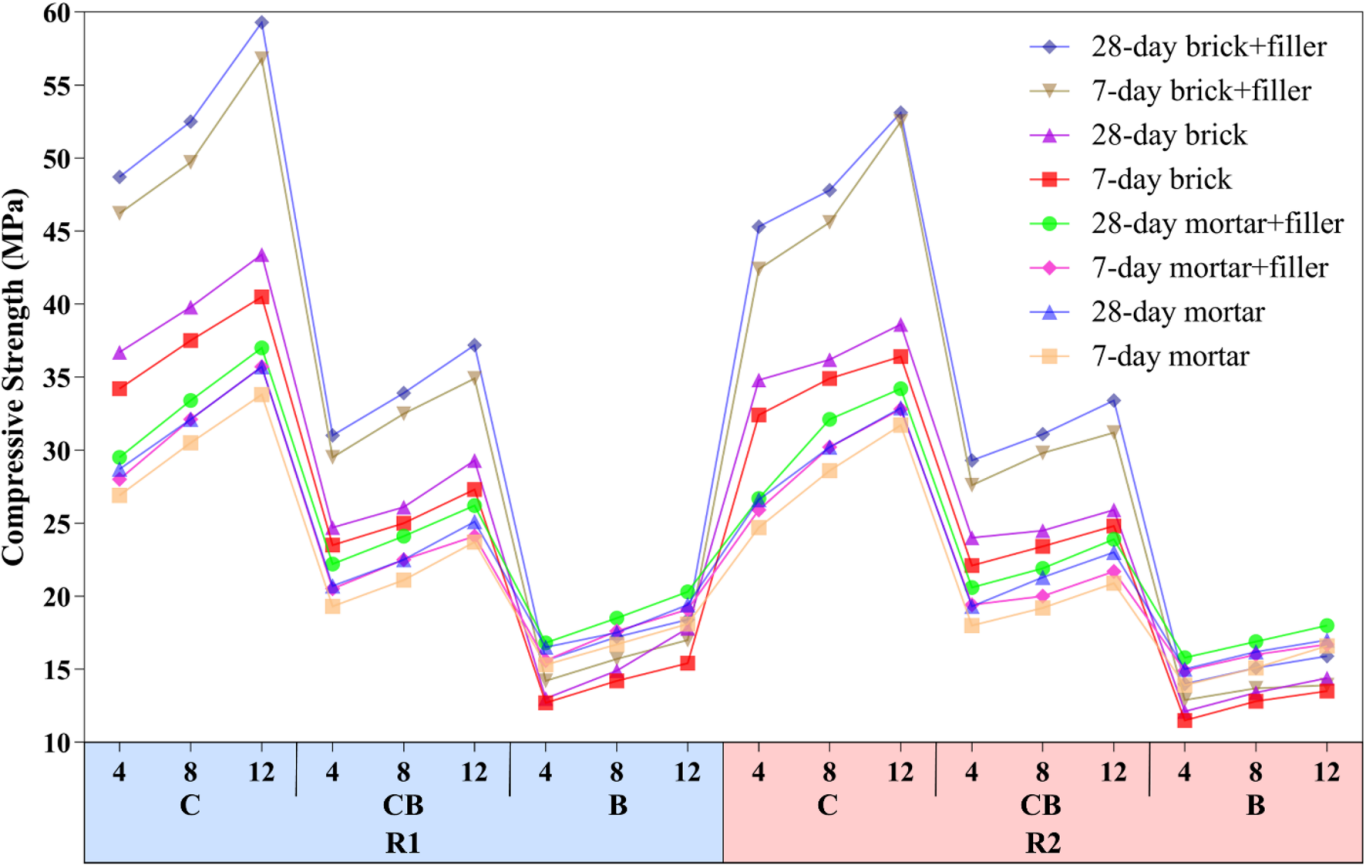
Compressive strength of the geopolymer samples.

This section evaluates the compressive strength of geopolymer samples with different mix designs listed in Table 2 to determine the mix design that provides the highest compressive strength while making optimal use of raw materials. Based on the results, the best mix design is ultimately used for subsequent flexural and tensile strength testing. Additionally, the impact of sand filler addition on mortar and brick specimens was investigated. Compressive strengths of 7- and 28-day geopolymer samples including mortar and brick are shown in Fig. 3. In general, Compressive strength was higher for R1 samples than for R2 samples. Furthermore, by comparing the different proportions of RCP and RBP in the geopolymer samples, it was found that samples composed entirely of RBP (B) had the lowest compressive strength, while samples composed entirely of RCP (C) had the highest values. Medium compressive strength was also demonstrated by samples prepared with mixed powder (CB). Therefore, it can be concluded that an increase in RCP, improves the compressive strength of the geopolymer samples. As a result, the compressive strength of 28-day filler-free mortar and brick samples with R1 and NaOH at a concentration of 12 was 43.4 and 35.7 MPa, respectively. When 50% sand filler was added to the mentioned samples, their compressive strength reached 59.3 and 37 MPa for mortar and brick, respectively, which is an increase of about 36.5% for brick and 3.5% for mortar. Since the same materials were utilized to prepare both geopolymer bricks and mortars, earlier research has demonstrated that, in the case where a filler is incorporated into the mortar mold, the size of the mold may significantly impact the compressive strength, which helps to explain the variation in compressive strength achieved. A larger mold can result in a greater distribution of stresses inside the mortar and a higher compressive strength since it improves the contact surface area between the filler and the mortar. Additionally, the mortar's compressive strength can be impacted by the mold's size. The ratio of the mortar's surface to volume decreases with mold size, which can result in more consistent and much better mortar curing and an improvement in the mortar's compressive strength^71–74^. This justifies the difference in compressive strength seen in mortar and brick with and without filler in the current study.

Moreover, all geopolymer samples showed an almost linear increase in compressive strength with a total increase of around 17–21% across all samples upon increasing the concentration of NaOH alkaline solution from 4 to 12 M. This is consistent with the results of previous studies that increasing NaOH concentration increases compressive strength^60,75^. NaOH is a decomposing agent in alkaline solutions that breaks down the molecules in pozzolans into atoms or their oxide components^76^, and WG acts as an adhesive in geopolymers^77^. Therefore, as the NaOH concentration in the geopolymer samples increased, so did the compressive strength. For a 28-day brick with filler and NaOH 12 M, increasing R1 to R2 reduced the compressive strength from 59.53 to 53.1 MPa. This 10–11% decrease was also observed in the compressive strength of all samples. The compressive strength of geopolymers is often reduced by an increase in the liquid/binder ratio resulting from the addition of more WG to the mix (see Table 2). For an effective bond to occur between the particles, the liquid/binder ratio should be within a suitable range^78–80^.

Geopolymer samples consisting of all RCP, all RBP, and a 50% mixture of both were examined to determine the influence of the different binder types on compressive strength. As Fig. 1 shows, the samples composed solely of RCP have a gray color, indicating that their binder is the waste concrete, while the orange color of the samples that consist only of RBP is related to the type of waste brick. The results presented in Fig. 3 show that the compressive strength of geopolymer samples decreases when the binder material is changed from RCP to RBP. Given that RCP and RBP have almost identical concentrations of SiO_2_ and Al_2_O_3_ (see Table 1), it is believed that the high Ca concentration in RCP is the cause of the increased compressive strength observed in samples prepared from RCP. Consequently, Ca plays an important role in providing a significant portion of the compressive strength achieved in the RCP samples, which subsequently occurs through the formation of CSH. This is consistent with what earlier research has shown^81,82^. By substituting RCP for RBP in the 28-day B-12-1 brick sample with filler, the compressive strength enhances from 18.4 to 59.3 MPa, indicating a 220% growth. Furthermore, in the mentioned sample, this number equals 103% if RCP is used in place of half of RBP. The results from all the samples produced for this study show that the increase in compressive strength that occurs from the addition of RCP in geopolymer samples is almost linear.

In addition to the aforementioned, the geopolymer samples' slight mechanical strength differences after 7 and 28 days show that they obtained a substantial portion of their strength in the early days of curing, which is because they were cured for 3 days at 80 °C in an oven.

**Figure 4:**
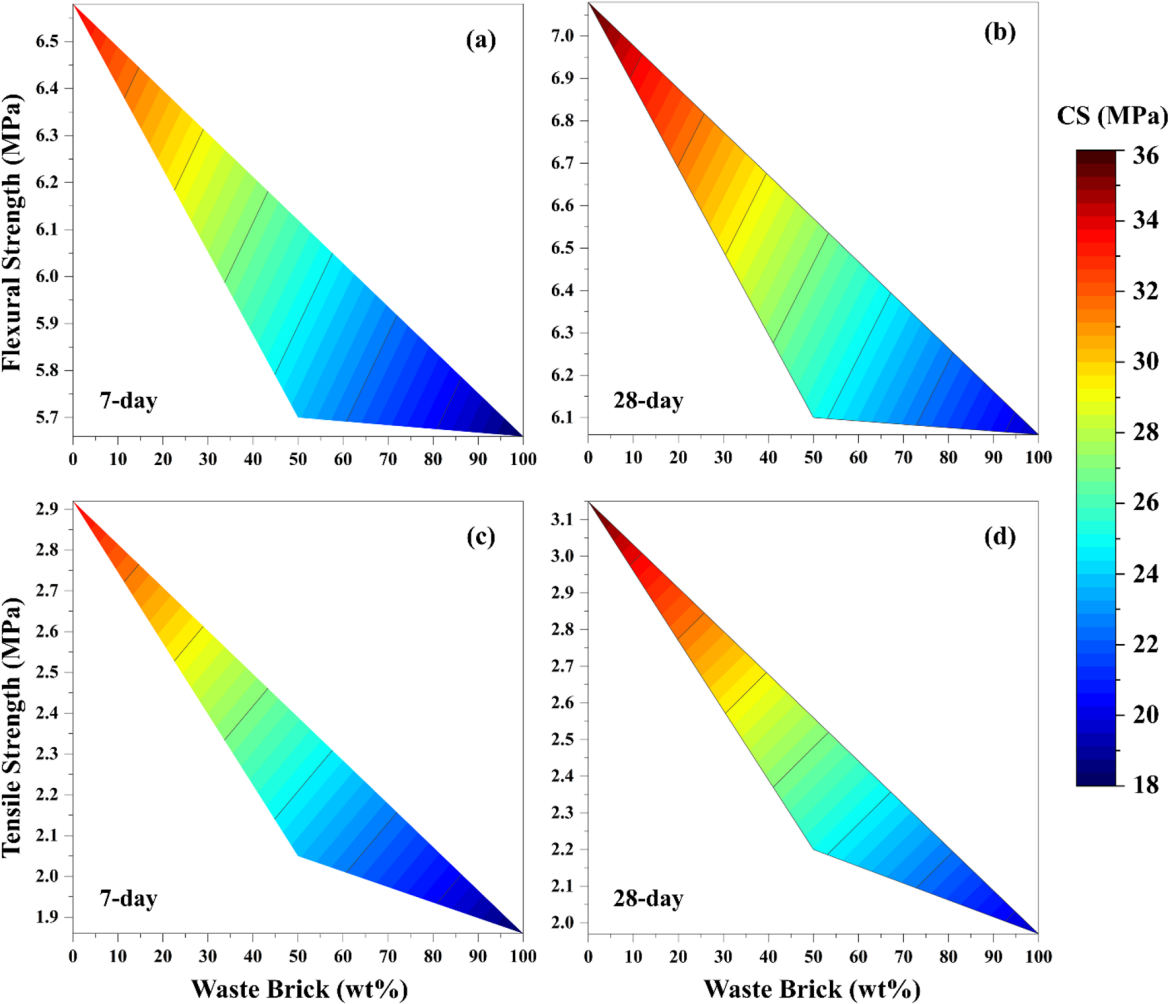
Flexural strength and tensile strength of free-filler geopolymer mortars with 12M and R1 (CS: compressive strength).

Given that the X-12-1 samples had the highest compressive strength, the samples of geopolymeric filler-free mortars were chosen to assess the tensile and flexural strengths to further investigate the behavior of the specimens. The contour graph in Fig. 4 illustrates the relation between compressive strength and flexural strength or tensile strength, highlighting the variations caused by the addition of RBP. In other words, Fig. 4 shows the simultaneous and combined analysis of the three variables—compressive strength, flexural or tensile strength, and RBP amount—as well as the interaction between them.

The flexural strength and tensile strength values in the 28-day samples with 50 wt% RBP (Fig. 4b and d) were 6.1 and 2.2 MPa, respectively. The flexural strength and tensile strength decreased to 6.06 and 1.97 MPa in response to increasing the RBP content to 100 wt%. In summary, there is a direct correlation between the addition of RCP ​​and the mechanical strength of specimens. Furthermore, taking into account that the majority of the mechanical strength was acquired in 3 days of curing in oven, the mechanical strengths of the 7- and 28-day samples are similar and slightly different from each other.

Considering that the tensile/compressive strength ratio of cement mortar typically ranges from 7 to 12% (with an average of around 10%). From the data presented in Fig. 4, it can be taken out that the 28-day geopolymer mortars prepared in this study had tensile/compressive strength ratios between 8.8 and 10.28%, indicating that they can be considered comparable to cement mortars.

**Figure 5:**
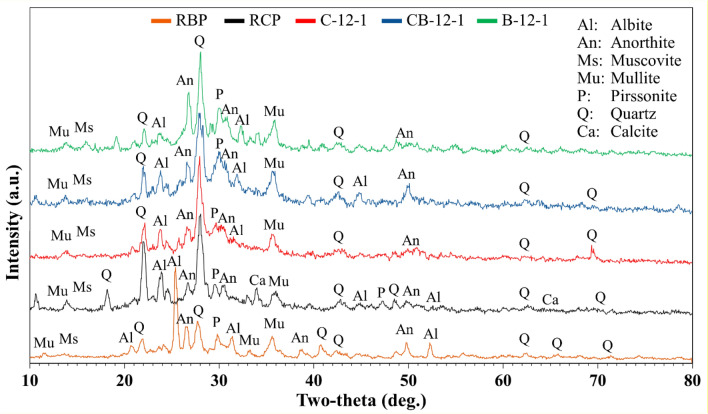
XRD spectrums of RBP, RCP, and geopolymer samples made with NaOH 12M and R1.

The XRD spectrums of the RBP, RCP, and 28-day samples with 12 M NaOH concentration and R1 are shown in Fig. 5. Since an alkaline solution was used, the peaks in the spectrum of RCP and RBP differ slightly from the peaks of the samples derived from them. Due to the uniformity of the alkaline solution in all samples, comparatively new peaks are formed during the geopolymerization reaction with the aluminosilicate sources in RCP and RBP. These peaks are almost identical.

Numerous crystalline phases, including muscovite, pirssonite, albite, anorthite, mullite, and quartz, were present in the semi-amorphous structure represented by the XRD spectra of the geopolymer samples. The results indicate a notable reduction in anorthite and albite's crystalline phases in comparison to RCP and RBP. Furthermore, since the quartz intensity of the samples was higher, the RBP quartz crystal phase did not contribute to the geopolymerization of the samples, whereas the RCP quartz crystal phase was consumed during the geopolymerization process. The quartz phase intensity in sample B-12-1 is higher than that in RBP, indicating the formation of quartz. This is because Si is present in high concentrations in RBP, which results in the formation of quartz due to elevated curing temperature and high concentration of alkaline solution. The samples showed almost identical crystalline phases, although samples with RBP had lower quartz concentrations than sample C-12-1, suggesting that the RBP samples had more amorphous geopolymer phases^83^. Additionally, the geopolymerization reaction mechanism responsible for the creation of gehlenite-based C-A-S-H can be linked to the rise in the anorthite peak, whilst the development of N(C)-A-S-H gels can be linked to the intensification of the albite crystal phase^84^. Although C samples had the greatest mechanical strength, CB and B samples had more amorphous structures, indicating a high rate of geopolymerization.

### Water absorption

The amount of water absorption was measured by examining mortar and brick samples. In addition, the effects of various parameters were studied, including the replacement of RBP and 50% sand filler. Along with examining all that mentioned above, the R-value and NaOH concentration were also examined to find out how an alkaline solution affects water absorption.

**Figure 6:**
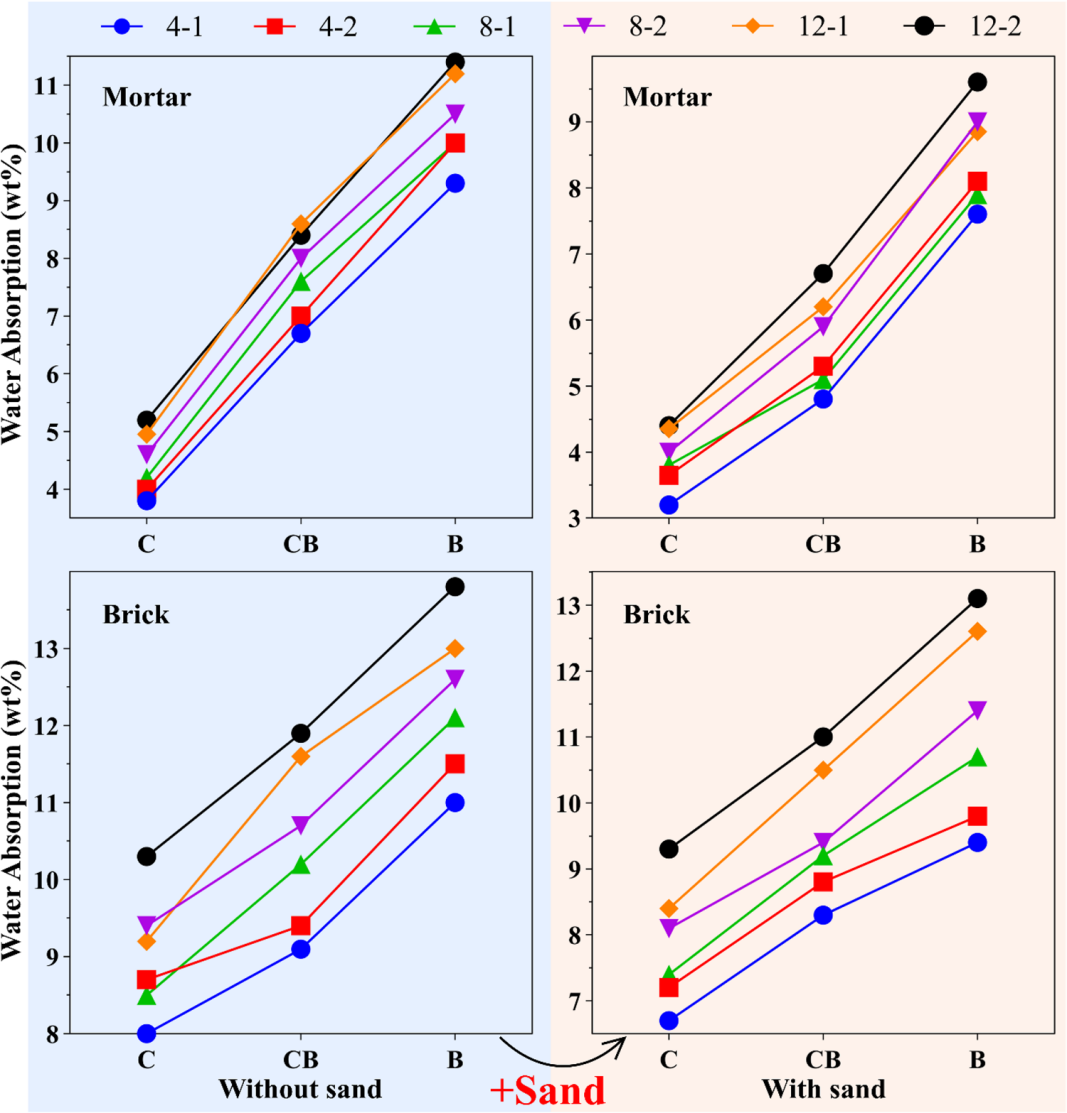
Water absorption and sorptivity of geopolymer bricks and mortars.

The outcomes of a 28-day water absorption test on geopolymer mortars and bricks are displayed in Fig. 6. Generally speaking, B samples absorbed the most water, whereas C samples absorbed the least. Thus, the average increase in water absorption in the mortar with and without filler was 50% and 75%, respectively, with the addition of 50 wt.% RBP. Higher RBP levels and other samples, such as brick, likewise exhibit this linear growth. The highest water absorption was obtained for the B-12-2 brick sample, which was equal to 13.8 wt.%. Also, the lowest water absorption was obtained for the C-4-1 mortar sample with sand filler, which was equal to 3.2 wt.%. This instance is consistent with earlier research, which has demonstrated in the literature that the RBP samples' porous nature and tiny pores increase water absorption^23,85^. Moreover, water absorption in mortar and brick samples is decreased by adding 50% sand filler. As a consequence, after adding sand filler, the water absorption of the B-12-2 mortar samples decreased by around 16%. This number also equals 12% for the mortar sample with the highest mechanical resistance, C-12-1. In addition to mortar, the addition of filler to B-12-2 brick samples reduced water absorption by about 5%, while it reduced water absorption by 8.6% in C-12-1 brick samples. In addition to the previously listed variables, water absorption increases linearly as R-value and NaOH concentration grow. This confirms the findings of the previous studies^86,87^.

### Flowability

The flow test on mortar was conducted to determine the flowability of the fresh mortar and to investigate the effects of varying amounts of alkaline solution, RCP, and RBP. It should be highlighted that an increase in l/b was necessary to control the mortar's flowability and enhance its efficiency (see Table 2). Due to RBP's high water absorption, some of the alkaline solution was utilized in the reaction with RBP. This considerably decreased flowability and efficiency and affected the mixing process.

**Figure 7:**
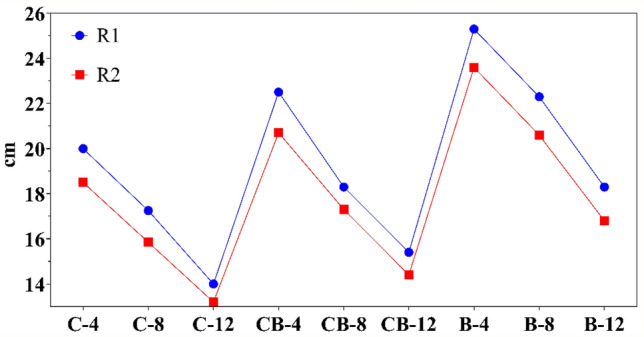
Flowability of filler-free mortar.

The data presented in Fig. 7 shows that the flowability of the mortar decreases as the amount of WG increases (from R1 to R2). In addition, the fluidity decreases significantly as the concentration of the alkaline solution increases. Samples C-4-2 and C-12-2 showed a drop in fluidity of 18.5 to 13.2 cm, or roughly 28%, with an increase in NaOH content. Furthermore, the mortar's fluidity was improved by adding RBP. In sample C-12-2, the addition of 50 wt.% RBP resulted in an increase of roughly 9% in the fluidity of mortar, which rose from 13.2 to 14.4 cm. Due to the nature of RBP, adding it to geopolymer mortar results in a rise in flowability with linear behavior. The findings of the EDX test show that RBP is mostly made of Si, whereas RCP is mostly made of Ca and Si (see Table 2). Ca therefore interacts with Si and Al to generate CSH and CASH structures and has a high reactivity rate in alkaline conditions^41^. Consequently, it is anticipated that samples containing RBP will have greater flowability.

### Applicability of bricks

In this work, the assessment of geopolymer bricks made from recycled materials is crucial for understanding their potential applications. As a result, their structural utility or applicability as paving was investigated. In terms of structural brick, as to ASTM C62-17^88^, Grade NW bricks have a minimum compressive strength of 8.6 MPa and are not limited in terms of water absorption. As a result, every brick sample included in this study is acceptable while evaluated just from the perspective of compressive strength. Moreover, the minimum compressive strength and maximum water absorption for Grade MW bricks are 15.2 MPa and 25 wt.%, respectively. Through comparison with the brick samples used in this study, the water absorption of each sample was confirmed. However, six samples—B-4-1 (without filler), B-8-1 (without filler), B-4-2 (with and without filler), B-8-2 (without filler), and B-12-2 (without filler)—did not qualify in terms of compressive strength; the remaining samples can be used as Grade MW structural bricks. Furthermore, the minimum compressive strength of 17.2 MPa and the maximum water absorption of 20 wt.% are applicable as Grade SW bricks. In light of this, all brick samples were allowed in terms of their water absorption, but in terms of their compressive strength, three more samples—B-4-1 (with filler) and B-8-2 (with filler) and B-12-2 (with filler)—were rejected in addition to the six previously rejected samples.

To maintain surface stability, thermal performance, and insulation and reduce water penetration into the structure, it is necessary to determine the maximum water absorption of bricks. These factors improve the functionality of bricks and increase their resistance to environmental impacts.

When it comes to paving, there are two scenarios in which the bricks are examined: one follows ASTM C902-22^89^ for pedestrian and light traffic, and the other follows ASTM C1272-22a^90^ for heavy vehicular paving. For pedestrian and light traffic, bricks with a compressive strength of more than 17.2 (Class MX & Class NX) and 48.3 MPa (Class SX) are taken into consideration; the results of Class MX & Class NX are identical to those of Grade SW, so all bricks with a compressive strength of more than 17.2 are approved (9 bricks were disqualified); however, only four brick samples for Class SX were approved, including C-4-1 & C-8-1 & C-12-1 (with filler) and C-12-2 (with filler). Furthermore, in accordance with ASTM C1272-22a, the bricks produced in this study are only appropriate for placement on a bituminous or concrete bed, which is also permitted for bricks with a compressive strength greater than 48.3 MPa. This is especially true if the bricks are intended to be used for vehicular paving. Consequently, only four samples—the same four samples from Class SX—were accepted out of the bricks prepared in the present study.

### SEM/EDX

**Figure 8:**
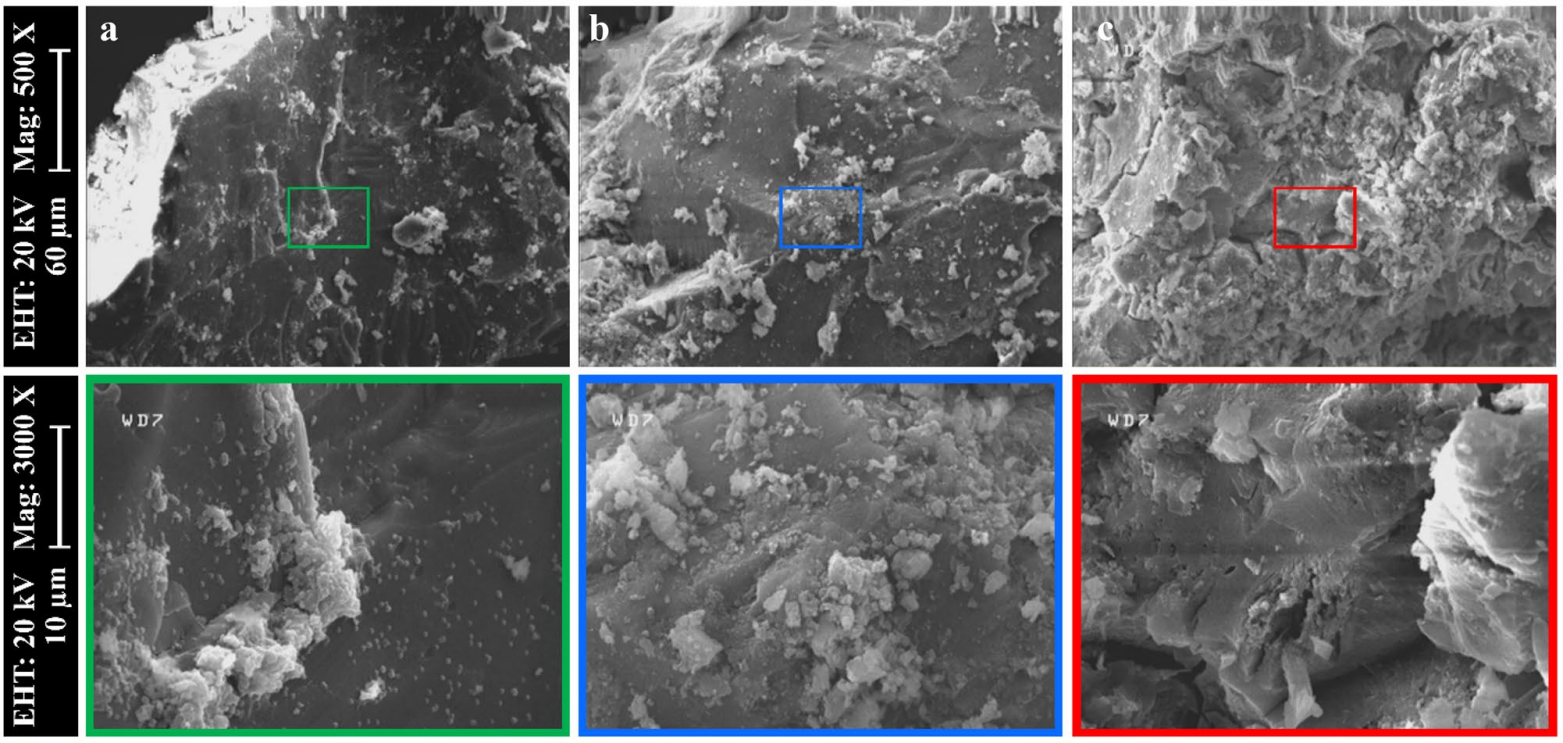
SEM images of the geopolymer samples a: C-12-1, b: CB-12-1, and c: B-12-1, showing details of scale, electron high tension, and magnification on the left side for each row.

SEM/EDX analysis was performed on the samples with the highest mechanical resistance (X-12-1) to evaluate the microstructure and understand the process of geopolymerization of the samples of the present study. Figure 8 shows SEM images of samples at 500 × and 3000 × magnification. The surfaces of samples C-12-1 (Fig. 8a) and CB-12-1 (Fig. 8b) were uniform, crack-free and smooth. In contrast, sample B-12-1 (Fig. 8c) exhibited many cracks and unreacted materials, which is consistent with the lower mechanical strength and higher water absorption of the B samples. Therefore, the matrix produced in samples with RCP had higher strength, which consequently provided a better surface.

In Table 2, Si/Al, Na/Si, Na/Al, and Ca/Si ratios for RBP, RCP, and X-12-1 samples are compared. (Si/Al)_RCP_ > (Si/Al)_RBP_, and (Ca/Si)_RCP_ > (Ca/Si)_RBP_ indicates that RCP is a rich source of Si and Ca. Geopolymers are classified into three types based on their Si/Al ratio^91^: a) poly(sialate): Si/Al = 1, b) poly(sialate-siloxo): Si/Al = 2, and c) poly(sialate-disiloxo): Si/Al = 3. Therefore, they have a three-dimensional to semi-crystalline structure^35,92^. In this study, structures of C-12-1 and B-12-1 samples were classified as poly(sialate-disiloxo) with a sialate link in it, whereas, the structure of the CB-12-1 sample was classified as poly(sialate-siloxo). Furthermore, from the SEM images of C and CB, it can be obtained that their smooth and uniform surface can be related to Ca. Since CHS gel can be generated during the geopolymerization process, resulting in a smooth surface. According to Table 2, the amount of Ca in samples of C and CB is substantially larger than in sample B, implying that (Ca/Si)_C_ or (Ca/Si)_CB_ > (Ca/Si)_B_. It should be noted that excess quantities of Na also result in a two-dimensional structure, an inhomogeneous surface, and poor durability^93,94^.

## Conclusion

In this study, new bricks and mortars were produced from waste bricks and waste concrete using geopolymer technology. For further investigation, the effects of NaOH concentration, WG/NaOH, and the addition and replacement of RBP instead of RCP were evaluated. In addition to the mechanical strength, the water absorption of bricks and mortar was also examined. Additionally, SEM/EDX and XRD analysis were used to investigate the microstructure and composition of geopolymer samples. The most important results of the findings of this work are as follows:

1. The mechanical strength of the samples increased with increasing amounts of RCP. In addition, variations in R-value and NaOH affected the compressive strength. Accordingly, the compressive strength increased with increasing NaOH concentration, and a slight decrease in the compressive strength of the samples was observed as R1 increased to R2.
2. Sand filler had a positive effect on the compressive strength of the samples. In such a way that the addition of filler increased the strength.
3. Out of all the samples, the highest compressive strength was obtained for R1 samples with 12 M of NaOH, and among these samples, C-12-1 brick with filler had the highest compressive strength of 59.53 MPa.
4. The tensile and flexural strengths of the geopolymer samples were both satisfactory. Tensile/compressive strength ratios in geopolymer mortar ranged from 8.8 to 10.28%, which is comparable to that of OPC mortar.
5. Water absorption increased linearly with increasing R-value and NaOH content. The filler-free brick sample of B-12-2 had the maximum water absorption (13.8 wt.%); the addition of filler decreased the water absorption by 5%.
6. The results of this study showed that according to different standards, both waste concrete and waste brick can be used to produce geopolymer bricks for construction and paving purposes.
7. Ca improves the samples' strength and surface smoothness, according to SEM/XRD and XRF analyses. Consequently, it lowers the absorption of water as a result of the elimination of voids and cracks.

These novel materials can be suitable for use as structural and construction materials. The materials obtained in this study have environmental and economic benefits. On the one hand, these materials are environmentally friendly and minimize the amount of waste and pollutants released into the environment through the consumption of waste bricks and waste concrete and preventing from consumption of natural resources. On the other hand, costs are reduced by recycling and creating new resources from CDW. Thus, in the event of natural disasters or extensive building destruction, it is feasible to create new bricks and concrete through the economical and environmentally sustainable recycling of CDW.

## Data Availability

The datasets used and analyzed during the current study available from the corresponding author on reasonable request.
